# A novel EPID‐based MLC QA method with log files achieving submillimeter accuracy

**DOI:** 10.1002/acm2.14450

**Published:** 2024-06-20

**Authors:** Chenlu Liu, Binbing Wang, Xue Bai, Xiaolong Cheng, Xiaotong Wang, Xiaohua Yang, Guoping Shan

**Affiliations:** ^1^ School of Nuclear Science and Technology University of South China Hengyang Hunan PR China; ^2^ Department of Radiation Physics Zhejiang Cancer Hospital Hangzhou Institute of Medicine (HIM) Chinese Academy of Sciences Hangzhou Zhejiang PR China

**Keywords:** EPID, full‐width half‐maximum, MLC calibration, MLC QA

## Abstract

The purpose of this study is to develop an electronic portal imaging device‐based multi‐leaf collimator calibration procedure using log files.

Picket fence fields with 2–14 mm nominal strip widths were performed and normalized by open field. Normalized pixel intensity profiles along the direction of leaf motion for each leaf pair were taken. Three independent algorithms and an integration method derived from them were developed according to the valley value, valley area, full‐width half‐maximum (FWHM) of the profile, and the abutment width of the leaf pairs obtained from the log files. Three data processing schemes (Scheme A, Scheme B, and Scheme C) were performed based on different data processing methods. To test the usefulness and robustness of the algorithm, the known leaf position errors along the direction of perpendicular leaf motion via the treatment planning system were introduced in the picket fence field with nominal 5, 8, and 11 mm. Algorithm tests were performed every 2 weeks over 4 months.

According to the log files, about 17.628% and 1.060% of the leaves had position errors beyond ± 0.1 and ± 0.2 mm, respectively. The absolute position errors of the algorithm tests for different data schemes were 0.062 ± 0.067 (Scheme A), 0.041 ± 0.045 (Scheme B), and 0.037 ± 0.043 (Scheme C). The absolute position errors of the algorithms developed by Scheme C were 0.054 ± 0.063 (valley depth method), 0.040 ± 0.038 (valley area method), 0.031 ± 0.031 (FWHM method), and 0.021 ± 0.024 (integrated method). For the efficiency and robustness test of the algorithm, the absolute position errors of the integration method of Scheme C were 0.020 ± 0.024 (5 mm), 0.024 ± 0.026 (8 mm), and 0.018 ± 0.024 (11 mm).

Different data processing schemes could affect the accuracy of the developed algorithms. The integration method could integrate the benefits of each algorithm, which improved the level of robustness and accuracy of the algorithm. The integration method can perform multi‐leaf collimator (MLC) quality assurance with an accuracy of 0.1 mm. This method is simple, effective, robust, quantitative, and can detect a wide range of MLC leaf position errors.

## 1 INTRODUCTION

Intensity‐modulated radiation therapy achieves optimal treatment dose distribution by superimposing a series of irregular treatment fields shaped by a multi‐leaf collimator (MLC) on the tumor, which generates a steep dose gradient around the tumor.^1^, ^2^, ^3^, ^4^, ^5^, ^6^, ^7^, ^8^, ^9^ The dosimetric advantages of intensity‐modulated radiotherapy are highly dependent on the positioning accuracy of the MLC.^8^, ^9^, ^10^, ^11^, ^12^ Many studies have shown that MLC leaf position errors can lead to incorrect dose delivery. The dosimetric consequences of leaf position errors would be more pronounced for treatment fields where the distance between opposing leaves is typically small.^4^, ^8^, ^13^, ^14^, ^15^ Rangel et al.^14^ reported that 1 mm MLC system positional errors can result in an average difference of 2.7% and 5.6% of the reference equivalent uniform dose for prostate and head and neck clinical target volumes, respectively. In another study, every 1 mm leaf position error occurred in the static MLC subfield abutments could lead to an average 16.7% dose error for 6 MV photons and 12.8% for 18 MV photons.^16^ Therefore, to ensure the accuracy of the dose delivered to the patient during each treatment, the performance of the MLC must be tested periodically.

In recent years, with the development of radiotherapy technology and the improvement of radiotherapy equipment, the quality assurance (QA) procedures for radiotherapy, including routine machine QA and treatment planning QA, have become laborious, complex, and time‐consuming.^17^, ^18^ Thus, the development of a simple, quantitative, efficient, accurate, and robust MLC performance evaluation technique can yield significant benefits for the daily QA test conducted by clinical physicists. The picket fence method, traditionally used with radiochromic film, is the current standard for MLC QA.^2^, ^11^, ^19^, ^20^, ^21^ It is reported that MLC leaf positioning accuracy has been evaluated by many institutions using visual inspection of high‐intensity regions in the film.^10^, ^19^, ^20^ However, visual inspection is subjective and difficult to quantify and explain. Scanning film is a potentially valuable alternative, but this method is still time‐consuming and labor‐intensive^2^, ^4^, ^11^ Moreover, the application of MLC QA methods, including the water tank method,^22^ the ionization chamber method,^23^ and the ionization chamber array method,^1^, ^19^, ^24^ is also limited by their time‐consuming nature and low spatial resolution.

The electronic portal imaging device (EPID) has been proven to be a powerful tool with the potential to replace the film for MLC QA tests.^4^, ^25^, ^26^ EPID was widely used for both patient and machine QA studies due to the advantages of providing digitized images and integration with routine linear accelerators. Currently, EPID‐based MLC positioning detection algorithms are successful, but they are not simple and fast enough, and the detection range is often limited to ± 1 mm.^2^, ^4^, ^5^, ^7^, ^26^, ^27^, ^28^, ^29^ Mamalui‐Hunter et al.^26^ suggested a way to determine the accuracy of MLC leaf positioning by using a modified Lorentzian function to fit the abutment peaks and a fourth‐order polynomial to make a calibration curve between the nominal gap width and the relative peak height. However, it took 30 min to analyze a strip test image with this algorithm. Li et al.^30^ showed that the relationship between full‐width half‐maximum (FWHM) and gap width had a linear turning point as the gap width increased. The authors developed a relatively simple algorithm for the linear part of the relationship but lacked a nonlinear one, and its MLC positioning error detection range was also limited to ± 1 mm. In addition, other authors noted that it was insufficient to only perform the picket fence test for MLC QA due to the instability of the pixel intensity values, so a nongap test was supposed to be supplemented.^11^ However, supplementary tests increase the workload of clinical physicists.

Log files were widely used in machine QA and patient‐specific QA due to the advantage of real‐time recording of accelerator performance information.^3^, ^6^, ^13^, ^15^, ^31^ They also applied to MLC QA as a part of overall system QA. The log file can record the leaf positions, gantry angles, and collimator angles in detail with high time resolution, which makes reviewing MLC performance at a later stage possible. In the current publication, the algorithms developed were usually limited to the data processing results of a single tool. Only a few reports work on the development of algorithms combined with log files. The purpose of this study is to develop an EPID‐based MLC QA algorithm by combining the advantages of log files with the work of Li et al.^30^ and Mamalui‐Hunter et al.^26^ This algorithm can detect MLC leaf position errors larger than 1 mm with higher efficiency and robustness.

## 2 MATERIALS AND METHODS

### 2.1 Materials

#### 2.1.1 MLC and EPID

In this study, data acquisition was performed on an Infinity linear accelerator (Elekta AB, Stockholm, Sweden) with the Agility MLC collimator system. The Agility collimator consists of 80 leaf pairs with a projected width of 5 mm per leaf and provides a maximum field size of 40 × 40 cm^2^ at the isocenter. The Agility collimator is slanted to minimize leakage between the leaves without a tongue‐and‐groove design. The 6‐MV photon mode was selected for all measurements. The Agility MLC leaf features digital control and dynamic guidance. The leaf position can be precisely determined by the Rubicon optical system mounted on the linear accelerator head. The system enables real‐time monitoring of the leaf position by capturing the infrared fluorescence generated by the ruby tips on the MLC leaf with an infrared camera.

The Infinity EPID system (iView GT, Elekta AB, Stockholm, Sweden) includes an amorphous silicon flat plate imager for megavoltage image acquisition. The source‐to‐detector distance (SDD) is 160 cm. The sensitive area of the EPID is 41 × 41 cm^2^, containing 1024 × 1024 pixels, which can be imaged up to 26 × 26 cm^2^ at the beam isocenter. The pixel pitch of this panel is 0.4 mm. Gain and offset corrections were applied to the pixel values of the EPID image. Moreover, the EPID's alignment, SDD, and isocenter positions were calibrated before each measurement. The 6‐MV photon mode was selected for all measurements.

#### 2.1.2 Log files

Infinity Linear Accelerator log files can be exported directly from the treatment control computer via an optional backup utility. Information on all aspects of the accelerator during beam delivery has been recorded in a file with the extension “.trf” (treatment record file) in binary format. In the log file, a record is generated every 40 ms. Each record includes the accelerator status, control points, dose rates, gantry angles, collimator angles, and the positions of every leaf, and so forth. Further information about the Elekta log files has been described in detail in previously published work.^6^

### 2.2 Methods

#### 2.2.1 Parsing Elekta log files

All data processing described in this paper was implemented in Python (version 3.11.5) using the open‐source Anaconda 64‐bit software (version 23.10.0), and the Python code was developed using the Pycharm (version 2023.2.5) integrated development environment. The acquired log files were sorted based on the time they were created and subsequently renamed. The initial binary format log files were converted to a .csv format by the pymedphys (version 0.38.0) Python toolkit. The leaf positions recorded by the log file for each segmentation were averaged and defined as actual leaf position. The abutment width parsed from the log file was defined as the actual abutment width.

#### 2.2.2 Stripe test design

**FIGURE 1:**
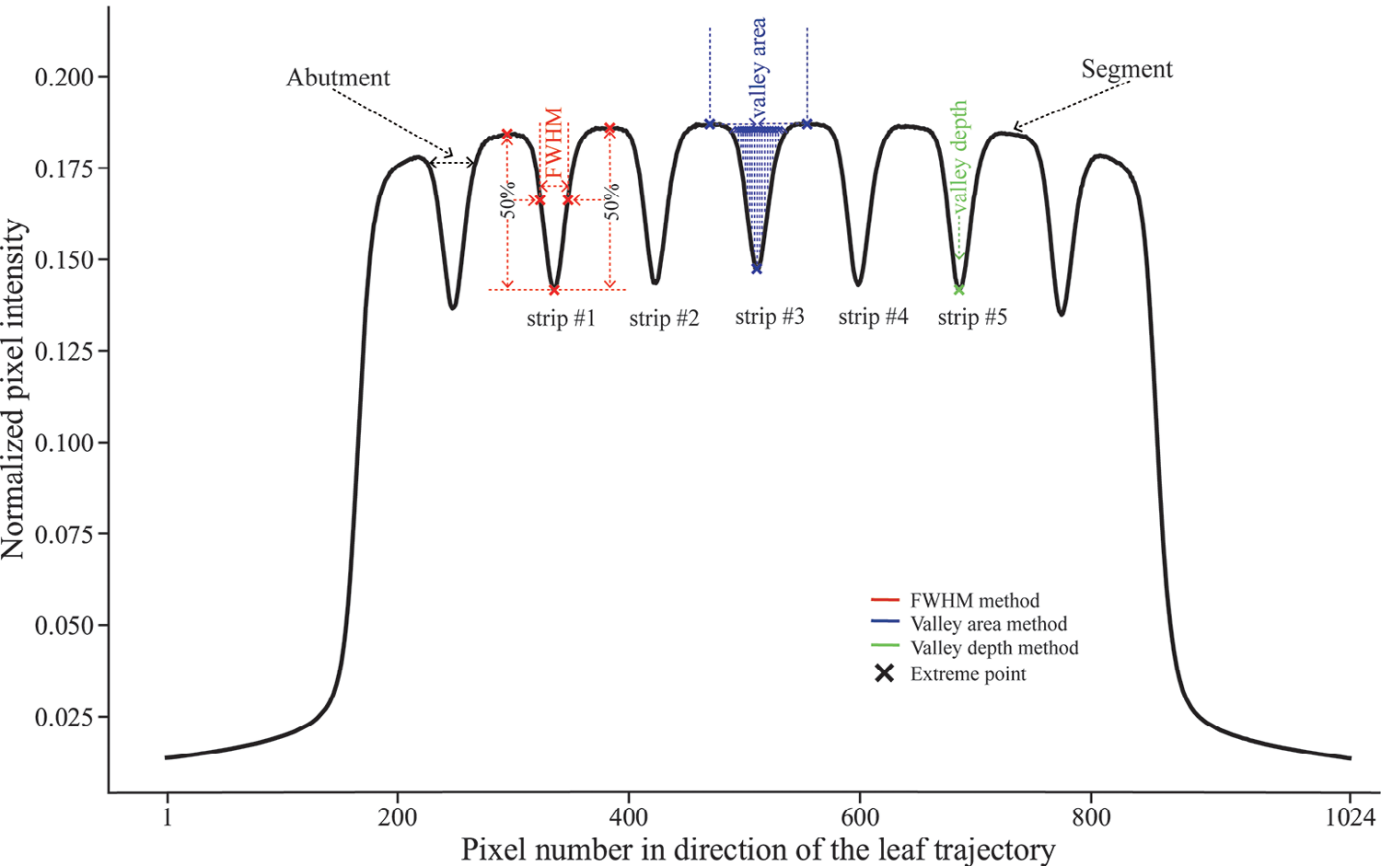
Normalized pixel intensity profile of a central leaf pair. This picture shows the basic principles of the three algorithms (FWHM method, valley area method, valley depth method) proposed in this study. FWHM, full‐width half‐maximum.

**FIGURE 2:**
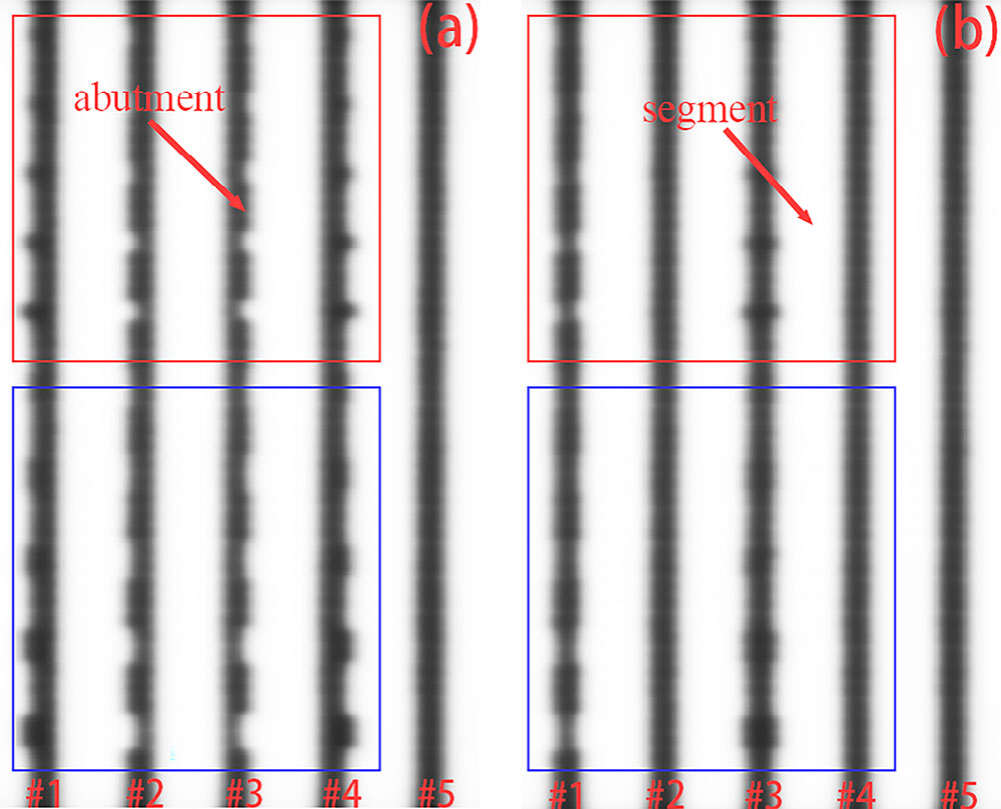
A schematic diagram of two positional error patterns introduced in leaf pairs with nominal width. Known positional errors are introduced for leaves of only one bank (a) and two banks (b). Each position error was assigned to a unique leaf pair in the region marked by the red box. Each position error was assigned to two consecutive adjacent leaf pairs in the region marked by the blue box. The white color represents the region where the EPID receives beam delivery (segment), and the black color is the opposite (abutment). EPID, electronic portal imaging device.

The picket fence test method proposed by Li et al.^30^ was used in this research. The picket fence test consisted of eight successive adjacent step‐and‐shoot segment irradiations 2 × 24 cm^2^ with an intentional gap specified by the Monaco treatment planning system (TPS, Elekta AB, Stockholm, Sweden, version 5.40.04), as shown in Figure 1. For expressive clarity and convenience, we defined this specified gap width as the nominal abutment width. Each picket fence test contained only a nominal abutment width. A total of 13 picket fence tests with nominal abutment widths ranging from 2 to 14 mm were designed and used to develop the MLC QA algorithm. It should be emphasized that the segment radiation on both sides only provided superimposed scattering for the experiment and did not participate in the subsequent data analysis. Ultimately, only five strips were used for the subsequent development of the MLC QA calibration algorithm (see Figure 1). Please note that the stripe here refers to the abutment region between two adjacent segment irradiations (see Figure 1). In other words, the strip was composed of abutment regions for all leaf pairs in the direction of perpendicular leaf motion, as shown in Figure 2. The dose delivery of 100 MU was performed for each segmentation using the 6 MV photon beam when both the gantry and collimator were set at zero degrees. Picket fence field tests with nominal strip widths were performed weekly for 2 months.

#### 2.2.3 Image data processing

EPID images were exported from the Mosaiq oncology management system (version 2.80 SP3) as DICOM files and imported for processing using the pydicom open source library (version 2.4.3). The amount of change in the pixel values for each detector caused by the dose delivery was obtained by subtracting the raw EPID image's pixel values from the number 65535. All processed picket fence field images were normalized by the 26 × 26 cm^2^ open‐field images acquired for each test to reduce the fluctuation of pixel values caused by beam energy variations. The corresponding formula was as follows:

(1) $$I_{norm}\;=\;\frac{\Delta I_{raw}}{\Delta I_{open}}$$

where $I_{norm}$ was the normalized image, $\Delta I_{raw}$ was the amount of change in the pixel value of the raw picket fence field image, and $\Delta I_{open}$ was the amount of change in the pixel value of the open field image.

#### 2.2.4 MLC QA algorithm development

To investigate the impact of different data processing methods on the accuracy level of the developed algorithms, three data processing schemes were performed in this trial. We used the log files to establish an independent calibration curve for each strip by averaging over all leaf pairs, which then defined this data processing method as Scheme A. In addition, Schemes B and C were defined as establishing an independent calibration curve for each leaf pair of each strip without and with log files, respectively.

In order to reduce the influence of interleaf leakage and enhance the robustness of the opposing leaf pairs data, the central 5 rows of strip image data in the direction perpendicular to the leaf trajectory were averaged. The averaged data was interpolated to a resolution of 0.01 mm using 3‐fold B‐spline interpolation and used as a baseline for subsequent analyses.

To fully exploit the information hidden in the picket fence images with nominal strip width used for algorithm development MLC QA instead of performing supplementary tests, we developed four MLC leaf positioning methods: the FWHM method, the valley area method, the valley depth method, and the integration method. In this study, we used the FWHM method proposed in a previously published article.^4^, ^5^, ^30^ The positions of the valley values and the peaks on both sides for the normalized intensity profiles across the abutment region were accurately determined by performing the ‘find_peaks’ function from the SciPy Python scientific computing library (version 1.11.3) on the interpolated data. The positions of the 50% values (peaks and valley values were averaged) on both sides of the normalized intensity profile were determined as the left and right boundaries. The boundary position can be obtained directly using a function called ‘where’ from the Numpy scientific computing library. The distance between the left and right boundaries was defined as FWHM, as shown in Figure 1. The FWHM calculation formula located in the abutment region (i,j) was defined as follows:

(2) $$FWHM_{i,j}\;=\;\frac{P_{i,j}^{r}\;-\;P_{i,j}^{l}}{100}$$

where 44, i = 1,…5, j = 1,…, r, l denoted stripe number, leaf pair number, right and left, respectively. P denoted the leaf position resolved from the picket fence field image used for the MLC QA algorithm development. To validate the accuracy of the FWHM method, a calibration curve characterizing the relationship between FWHM and abutment width was established. The FWHM of each leaf pair was the average of eight picket fence field tests as described in Section 2.2.2 before establishing the relationship curves. This FWHM was defined as the nominal leaf pair FWHM. Similarly, the average of the leaf positions and the average of the abutment widths parsed from the log files of the eight picket fence field tests were defined as the nominal actual leaf position and the nominal actual leaf pair abutment width, respectively. For a relationship curve, 13 data points were fitted with a fifth‐order polynomial. The corresponding formula was as follows:

(3) $$\begin{array}{ccc} W^{Abu}\; & = & \;c_{0}\;+\;c_{1}{\left(W^{F}\right)}^{1}\;+\;c_{2}{\left(W^{F}\right)}^{2}\\ & & +\;c_{3}{\left(W^{F}\right)}^{3}\;+\;c_{4}{\left(W^{F}\right)}^{4}\;+\;c_{5}{\left(W^{F}\right)}^{5} \end{array}$$

where ${\left\{c_{k}\right\}}_{k\;=\;0,\text{…},5}$ represented the coefficients of the 5‐fold polynomial. Here, $W^{A}$ and $W^{F}$ represented different meanings for different data processing schemes. For Scheme A, $W^{A}$ and $W^{F}$ represent the average of all nominal actual leaf pair abutment widths for individual strips parsed from the log file and the average of all nominal leaf pair FWHMs for individual strips from the picket fence image, respectively. For Scheme B, $W^{A}$ and $W^{F}$ represented the abutment width of each leaf pair of each strip specified in TPS and the nominal leaf pair FWHM of each leaf pair of each strip, respectively. For Scheme C, $W^{A}$ and $W^{F}$ represented the nominal actual leaf pair abutment width of each leaf pair of each strip and the nominal leaf pair FWHM of each leaf pair of each strip, respectively.

The valley area method was the area surrounded by the three successive adjacent extreme points (left peak height, valley value, and right peak height) of the normalized pixel intensity curve of the abutment (Figure 1). To simplify the algorithm, we directly used the absolute value of the sum of the differences between the data on both sides of the valley and the corresponding left and right peak values as the valley area, as shown in Figure 1. The valley depth method only took the valley value as the observed data used for function fitting (Figure 1). The data fitting process for the valley area and valley depth methods remained consistent with the FWHM method. Therefore, it was not described again here. The integration method was a method that comprehensively analyzes the outputs of the three independent algorithms described above at each test. Taking the absolute position error as the criterion, the value with the smallest absolute value among the results of the three independent algorithms was taken as the output of the integrated method and defined as the position accuracy of the integration method.

In this research, we used a method similar to Li et al.^30^ to determine the leaf positions in the picket fence field test images. The corresponding formula was as follows:

(4) $$P_{l}\;=\;P_{peak}\;-\;\frac{W}{2}$$

(5) $$P_{r}\;=\;P_{peak}\;+\;\frac{W}{2}$$

where $P_{l}$, $P_{r}$, $P_{peak}$ and W denoted the left leaf positions for the abutment region, the right leaf positions for the abutment region, the peak position for the abutment region, and the abutment width, respectively.

#### 2.2.5 MLC QA algorithm testing

To verify the accuracy of the algorithm proposed in this study, known leaf‐pair position errors of ±0.6, ±0.8, ±1.2, ±1.8, and ±2.4 mm were introduced in the TPS along the direction perpendicular to the leaf motion for the picket fence field with nominal strip widths of 5, 8, and 11 mm, respectively. Each known position error was assigned to a separate leaf pair and two consecutive adjacent leaf pairs within the common strip, as shown in Figure 2. The leaf pair position error experiment was designed with two test patterns, one for the single‐side bank leaf setting (Figure 2a) and the other for the combination of A and B bank leaf settings (Figure 2b). The measured leaf positions were compared against the actual leaf positions extracted from the log files. Leaf position error tests were performed once every 2 weeks in the 4‐month longitudinal evaluation.

#### 2.2.6 Statistical analysis

To evaluate the usefulness of the algorithm, the results of algorithm testing experiments for three picket fence fields with nominal abutment width (5, 8, and 11 mm) were compared and analyzed.

## 3 RESULTS

### 3.1 Analysis results of log files

**FIGURE 3:**
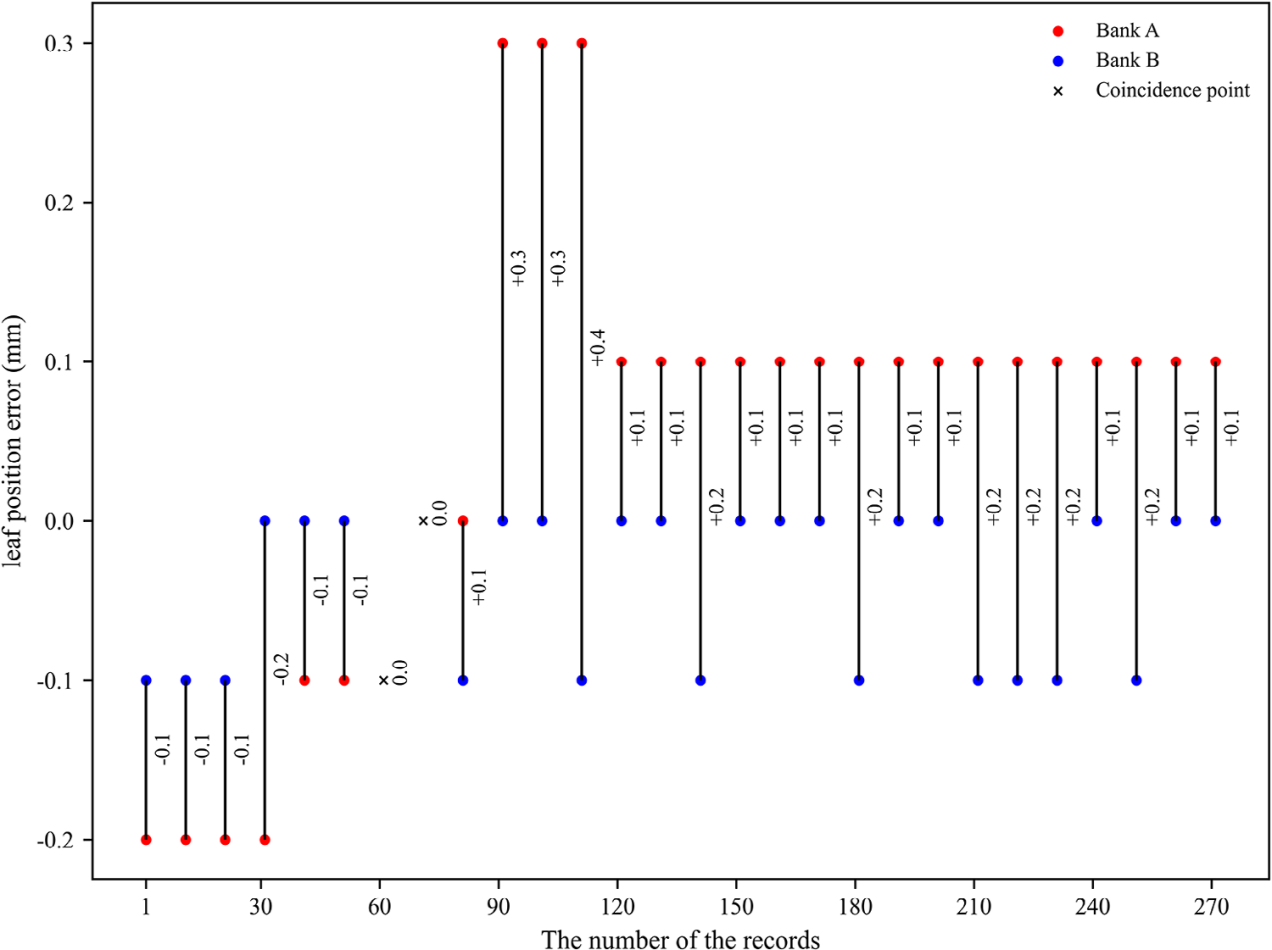
A schematic diagram of the variation of the positional error of the leaf pair and the abutment width error during a 100 MU dose delivery. To present the results clearly, we selected one to display every 10 records. + indicated the actual abutment width was larger than expected, − indicated the actual abutment width was smaller than expected.

According to the log file, approximately 272 rows of useful records were generated for each 100 MU dose delivered. In these records, we found that the leaf position was not always stable but jittered or bounced back and forth during dose delivery. To illustrate this point, we randomly selected the records of a leaf pair during a single dose delivery, as shown in Figure 3. The errors of the absolute MLC leaf position and the absolute abutment width created by the opposing leaf pairs were 0.054 ± 0.048 mm (mean ± standard deviation) and 0.077 ± 0.059 mm (mean ± standard deviation), respectively. The information about the leaf position errors and abutment width errors obtained from the log files was summarized in Table 1. Moreover, the probability of the occurrence of leaf position errors exceeding ± 0.1 and ± 0.2 mm in this work was approximately 17.628% and 1.060%, respectively. The occurrence of abutment width errors beyond ± 0.1 and ± 0.2 mm was approximately 29.111% and 4.219%, respectively.

**TABLE 1:** The error results of the leaf position and abutment width in the log file.

Category	Mean	SD	Max
Leaf position error (mm)	−0.033	0.064	0.250
Abs leaf position error (mm)	0.054	0.048	0.371
Abutment width error (mm)	0.003	0.097	0.361
Abs abutment width error (mm)	0.077	0.059	0.429

Abbreviations: Abs, absolute; Max, maximum; SD, deviation.

### 3.2 Accuracy of the algorithm

**FIGURE 4:**
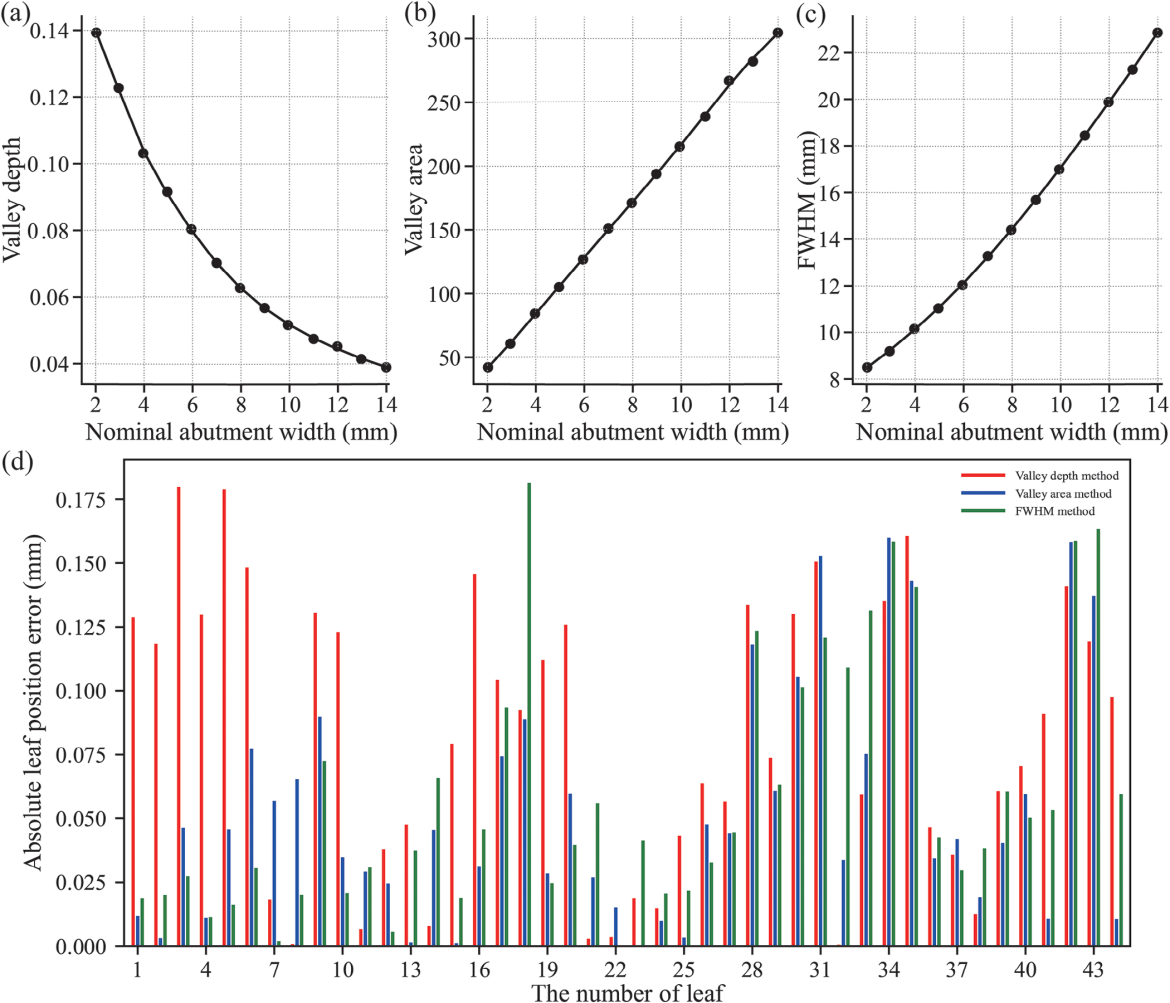
A comparison diagram of the calibration curves of the three algorithms developed by Scheme C for a leaf pair (a)–(c) and their accuracy for leaves of a single side bank for one strip (d).

The calibration curves of one leaf pair of different algorithms developed according to Scheme C were shown in Figure 4. The calibration curves of the valley depth method (Figure 4a) and the FWHM method (Figure 4c) showed nonlinear variations as the nominal neighbor width increased, and the turning points of their curves were 4 and 8 mm, respectively. In contrast, the calibration curve of the valley area method (Figure 4b) showed a linear variation. In addition, the three algorithms proposed in this work showed different levels of accuracy. The priority of the accuracy of the three algorithms was not fixed for all the leaves within a strip, as shown in Figure 4d.

**FIGURE 5:**
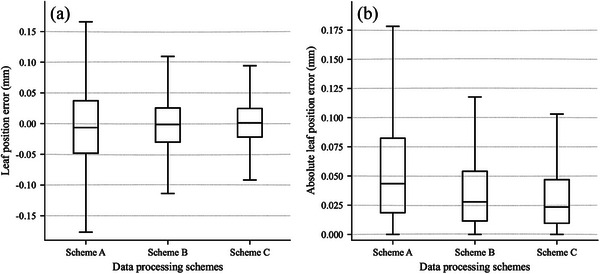
Results of the accuracy of algorithms developed by different data processing schemes. (a) Statistics of leaf position errors, (b) statistics of absolute leaf position errors.

There are differences in the level of accuracy of algorithms developed according to different data processing schemes, as shown in Figure 5. The algorithm developed according to Scheme C showed an optimal level of accuracy in terms of position error (Figure 5a) and absolute position error (Figure 5b) compared to other data processing schemes. The position accuracy, absolute position accuracy, and maximum absolute positional accuracy of the algorithm developed by Scheme C were 0.000 ± 0.057, 0.037 ± 0.043, and 0.522 mm, respectively. The position accuracy of other schemes was summarized in Table 2.

**TABLE 2:** The summary of the accuracy of the algorithms developed by different data processing schemes (A, B, C).

	Scheme A	Scheme B	Scheme C
Mean (mm)	−0.001	−0.002	0.000
SD (mm)	0.092	0.061	0.057
Mean Abs (mm)	0.062	0.041	0.037
SD Abs (mm)	0.067	0.045	0.043
Max Abs (mm)	0.695	0.533	0.522

Abbreviations: Abs, absolute; Max, maximum; SD, deviation.

Table 3 presented the position errors and absolute position errors of each algorithm in different data processing schemes. The errors of all algorithms followed a common pattern across different data processing schemes when the absolute position error was used as an evaluation criterion: integration method < FWHM method < valley area method < valley depth method.

**TABLE 3:** The results of the accuracy of the algorithms developed by the three data processing schemes (A, B, C).

Scheme	Valley depth	Valley area	FWHM	Integration
A				
Mean (mm)	0.002	−0.010	0.008	−0.003
SD (mm)	0.150	0.070	0.064	0.046
Mean Abs (mm)	0.110	0.055	0.050	0.034
SD Abs (mm)	0.102	0.044	0.041	0.032
Max Abs (mm)	0.695	0.320	0.357	0.229
B				
Mean (mm)	−0.005	−0.012	0.006	0.003
SD (mm)	0.085	0.059	0.050	0.037
Mean Abs (mm)	0.057	0.045	0.037	0.025
SD Abs (mm)	0.064	0.040	0.034	0.028
Max Abs (mm)	0.533	0.348	0.337	0.282
C				
Mean (mm)	−0.003	−0.010	0.009	0.003
SD (mm)	0.083	0.054	0.043	0.032
Mean Abs (mm)	0.054	0.040	0.031	0.021
SD Abs (mm)	0.063	0.038	0.031	0.024
Max Abs (mm)	0.522	0.278	0.346	0.211

Abbreviations: Abs, absolute; FWHM, full‐width half‐maximum; Max, maximum; SD, deviation.

### 3.3 Robustness of the algorithm

**FIGURE 6:**
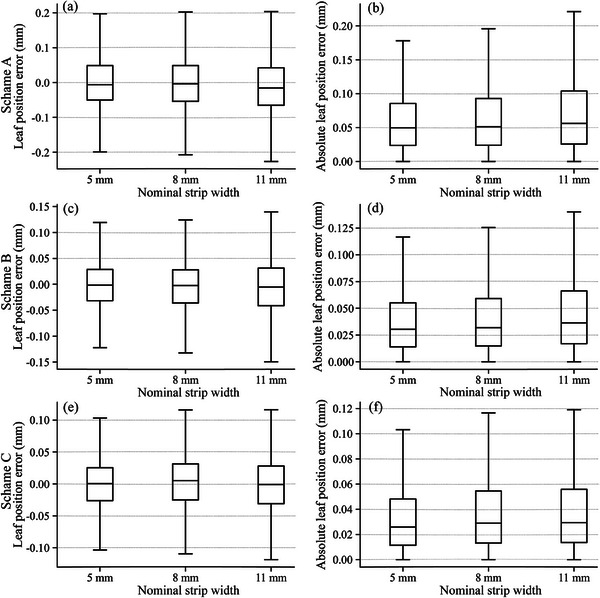
Results of the accuracy of three picket fence field error experiments with nominal strip width for Scheme A (a)–(b), Scheme B (c)–(d), and Scheme C (e)–(f).

In all the different data processing schemes, the range of position errors of the three independent algorithms (valley depth, valley area, and FWHM) tended to become larger as the nominal abutment width increases, as shown in Figure 6. The position errors of the three independent algorithms developed by Scheme C for picket fence field error experiments with nominal strip widths of 5, 8, and 11 mm were 0.000 ± 0.056, 0.000 ± 0.062, and −0.003 ± 0.070 mm, respectively. The corresponding absolute position errors were 0.038 ± 0.041, 0.042 ± 0.045, and −0.046 ± 0.053 mm, with maximum absolute position errors of 0.405, 0.470, and 0.522 mm, respectively. The results of the robustness of the independent algorithms based on other data processing schemes were summarized in Table 4. Furthermore, the robustness results of each algorithm for all schemes were summarized in Table 5.

**TABLE 4:** A summary of robustness test results for three algorithms (valley depth, valley area, FWHM) developed by different data processing schemes.

	Scheme A			Scheme B			Scheme C		
	5 mm	8 mm	11 mm	5 mm	8 mm	11 mm	5 mm	8 mm	11 mm
Mean (mm)	0.000	0.001	0.000	0.000	−0.006	−0.005	0.000	0.000	−0.003
SD (mm)	0.079	0.094	0.128	0.060	0.065	0.075	0.056	0.062	0.070
Mean Abs (mm)	0.061	0.069	0.085	0.042	0.045	0.052	0.038	0.042	0.046
SD Abs (mm)	0.051	0.064	0.096	0.043	0.047	0.054	0.041	0.045	0.053
Max Abs (mm)	0.443	0.533	0.695	0.394	0.478	0.533	0.405	0.470	0.522

Abbreviations: Abs, absolute; FWHM, full‐width half‐maximum; Max, maximum; SD, deviation.

**TABLE 5:** Results of the robustness of all algorithms based on different data processing schemes.

Method	Scheme A			Scheme B			Scheme C		
	5 mm	8 mm	11 mm	5 mm	8 mm	11 mm	5 mm	8 mm	11 mm
Valley depth									
Mean (mm)	−0.015	−0.002	0.022	−0.016	−0.011	0.012	−0.016	−0.006	0.015
SD (mm)	0.094	0.133	0.200	0.065	0.084	0.101	0.062	0.081	0.099
Mean Abs (mm)	0.076	0.102	0.152	0.047	0.056	0.068	0.042	0.054	0.067
SD Abs (mm)	0.057	0.085	0.132	0.048	0.063	0.076	0.048	0.061	0.074
Max Abs (mm)	0.443	0.533	0.695	0.394	0.478	0.533	0.405	0.470	0.522
Valley area									
Mean (mm)	−0.002	−0.002	−0.024	−0.002	−0.008	−0.028	−0.002	−0.002	−0.025
SD (mm)	0.068	0.069	0.067	0.055	0.058	0.061	0.051	0.055	0.053
Mean Abs (mm)	0.054	0.054	0.056	0.040	0.043	0.052	0.036	0.039	0.044
SD Abs (mm)	0.042	0.043	0.045	0.037	0.040	0.042	0.036	0.037	0.040
Max Abs (mm)	0.311	0.320	0.282	0.321	0.348	0.282	0.252	0.278	0.256
FWHM									
Mean (mm)	0.0159	0.007	0.002	0.017	0.001	0.001	0.016	0.007	0.002
SD (mm)	0.069	0.063	0.059	0.054	0.047	0.046	0.048	0.042	0.036
Mean Abs (mm)	0.053	0.049	0.047	0.038	0.036	0.036	0.034	0.032	0.027
SD Abs (mm)	0.047	0.039	0.037	0.041	0.031	0.029	0.038	0.027	0.025
Max Abs (mm)	0.357	0.282	0.264	0.337	0.304	0.282	0.346	0.234	0.210
Integration									
Mean (mm)	0.001	−0.002	−0.009	0.004	0.001	0.003	0.004	0.006	0.001
SD (mm)	0.047	0.047	0.044	0.037	0.040	0.035	0.031	0.035	0.030
Mean Abs (mm)	0.033	0.034	0.033	0.024	0.027	0.022	0.020	0.024	0.018
SD Abs (mm)	0.033	0.033	0.031	0.028	0.029	0.027	0.024	0.026	0.024
Max Abs (mm)	0.226	0.229	0.206	0.258	0.282	0.282	0.190	0.210	0.210

Abbreviations: Abs, absolute; FWHM, full‐width half‐maximum; Max, maximum; SD, deviation.

## 4 DISCUSSION

In this paper, we proposed an MLC QA algorithm to quantify the MLC leaf position during step‐and‐shoot IMRT delivery. The method combined the leaf position obtained from the log file to mine the information hidden in the EPID image. Subpixel resolution was achieved through cubic B‐spline interpolation, while the complexity of the algorithm was reduced by performing pixel‐by‐pixel comparison rather than analytic fitting. Furthermore, the integration method improves the accuracy and robustness of the algorithm by combining the advantages of three independent algorithms.

Current published MLC QA algorithms were usually developed based on a single tool. The accuracy of leaf positions was strictly dependent on the MLC calibration before the measurement,^10^, ^26^ which might make us ignore the occurrence of random leaf position errors during dose delivery. The leaf position error and abutment width error not less than ± 0.1 mm in this survey were about 17.628% and 29.111%, respectively. Therefore, it is crucial to pay close attention to the determination of the actual position of the leaf during the development and the test of the MLC QA algorithm. Failure to do so may result in making inappropriate conclusions. In this investigation, we fully considered the influence of the positional uncertainty due to leaf jitter during dose delivery. The actual leaf pair positions and abutment widths were determined by averaging all the records of individual dose deliveries in the log file, rather than the prescribed value. In this study, the accuracy of each of the algorithms (Table 3) developed by Scheme C, as well as their overall accuracy (Figure 5, Table 2), was better than that of Scheme B. This suggested that the log file is an effective tool to help develop an EPID‐based MLC QA algorithm. Besides, some studies have shown that the uncertainty of leaf position obtained from the log file is smaller compared to EPID.^3^, ^27^ In previous studies, leaf positioning strictly depended on MLC calibration before testing. However, MLC calibration does not guarantee the nonoccurrence of random leaf position errors during algorithm development and validation. And MLC calibration increases our workload as well. For that reason, the algorithm developed in this research was scientific, rigorous, reliable, and time‐saving.

In the present work, we established an FWHM calibration curve (Figure 4c) similar to that of Li et al.^30^ and also noticed that the nonlinear‐linear turning point of this calibration curve occurred around 8 mm. This might be explained by the fact that the valley value of the profile of the abutment region tended to saturate as the width of the abutment region increases (Figure 4a). Consequently, the FWHM magnitude depends mainly on the positions of the peaks on both sides (Figure 1). However, the size of the fitted data in the valley area method depends on more data points compared to the valley depth method and the FWHM method. And the closer to the peak position, the smaller the contribution the data points make to the valley area value. Thus, the valley area method is not sensitive to the fluctuation of the peak positions on both sides, which may be an important reason for the overall linear behavior of the calibration curve of the valley area method. Another important reason is that the fluctuation of pixel‐valued intensity caused by the beam energy variation is reduced through an open‐field normalization. The different behaviors exhibited between the algorithms for abutment width variations may explain their level of accuracy to some extent (Figure 4d). Compared to previous studies with abutment widths within 2 mm,^3^, ^4^, ^26^, ^32^ this study has a large abutment width (2–14 mm), which makes it possible to detect leaf position errors greater than 1 mm. Although Li et al.^30^ proposed the idea of detecting MLC leaf position errors in a relatively large range of abutment widths, the algorithm developed by them can only be implemented on abutment widths greater than 8 mm, whereas the algorithm developed in this study with a positional accuracy of 0.1 mm applies to 2–14 mm abutment widths.

Currently, there is no standardized method for the processing of MLC leaf strip data for picket fence fields. Mamalui‐Hunter.^26^ and Li et al.^30^ established calibration curves by averaging the data for each strip and all strips, respectively. While, Yang et al.,^10^ Sumida et al.,^11^ and Sakaria et al.^27^ used leaf‐by‐leaf pair analysis. This study showed that the position error, absolute position error, and maximum absolute error of each algorithm developed by Scheme A (Table 3), and their overall results (Table 2, Figure 5), were higher than those of Scheme C. This might be because the differences between the normalized pixel intensity profiles between each leaf pair were obscured by the average value. The work conducted by Mamalui‐Hunter et al.^26^ demonstrated that the pixel intensity profiles of the same leaf‐pair gap at different positions were not the same. Therefore, different data processing methods may affect the accuracy of the developed algorithms. In addition, the algorithm proposed by Li et al.^30^ did not adequately consider the differences in the results between different leaf pairs. Mamalui‐Hunter et al.^26^ considered the differences between the results of different strips in the direction of the leaf motion and established a calibration curve for each strip. However, the authors did not consider the differences between the results of different leaf pairs in the direction perpendicular to the leaf motion. In this paper, a calibration curve was established for each leaf pair of each strip, taking into account the differences between the leaf pair profiles. Thereby, our algorithm was personalized.

The algorithm's sensitivity may affect its accuracy. As the abutment width increases, the valley value of the valley depth method tended to saturate (Figure 4), and the accuracy decreased gradually (Table 5). Conversely, the FWHM method showed the opposite behavior to the valley depth method in terms of sensitivity and accuracy. The sensitivity and accuracy of the valley area method were approximately stable. Therefore, we found that the algorithm's accuracy was higher in the sensitive abutment width region. In addition, we also discovered that the overall accuracy of the three independent algorithms decreases as the abutment width increases (Figure 6, Table 4). Comparing Tables 4 and 5, we could see that the decrease in the overall accuracy of the algorithm was mainly caused by the valley depth method. Hence, we needed to perform the integration method. In this research, the integration method showed high accuracy and robustness to abutment width variations by integrating three independent algorithms (Table 5). In addition, both Sastre‐Padro et al.^2^ and Sumida et al.^11^ found that the pixel values at the abutment region were not stable. Sastre‐Padro et al.^2^ solved this problem by adopting a relative dose approach at the abutment region. However, in this work, we used open fields to normalize the EPID images, which made it much simpler to conduct experiments and develop algorithms. Sumida et al. used a nongap test as a supplement to the FWHM method, which increased the test time and physical cost. This did not fundamentally solve the problem. Furthermore, we developed methods apart from the FWHM method by fully exploiting the information in the strip test images, which did not require additional tests. The other methods developed in this work also played a complementary role to the FWHM method. The integration method especially took advantage of the strength of each independent algorithm and significantly improved the accuracy and robustness of this calibration procedure (Table 5).

Currently, the dominant EPID‐based MLC QA method is still the FWHM method. In all data processing schemes, the accuracy of the algorithms all followed a common pattern: integration method > FWHM method > valley area method > valley depth method, which had been proven in Table 3. However, the FWHM method was not always superior to the other two independent algorithms (Figure 4d). Therefore, The valley area and valley depth methods can be regarded as important supplements to the FWHM method. The stability of the valley area method and the sensitivity of the valley depth method could be utilized in clinical practice, but attention should be paid to the variation patterns of their respective accuracies in different abutment width ranges, as shown in Table 5. However, the integration method could avoid repetitive MLC QA tests due to the failure of a single algorithm. Finally, compared to the methods proposed by Li et al.^30^ and Mamalui‐Hunter et al.,^26^ each method in this work was developed by pixel‐by‐pixel comparison rather than function fitting. This significantly reduced the computational cost of the algorithms. Thus, the algorithms proposed in this study were effective and simple.

In addition, the data used for algorithm development in this paper was acquired weekly, and each acquisition was normalized by the open field taken separately for each measurement, which takes into account the small variations in flatness and symmetry of the beam energy and output in both in‐plane and cross‐plane directions. Conversely, the advantage of personalization provided by the algorithms developed according to Scheme C compared to Scheme A would encounter the risk of loss if we do not consider these variations. Therefore, a feasible approach is to reduce the impact of these variations on the generation of the algorithm by incrementing additional data and averaging them, but this approach extends the period of data acquisition. In theory, the algorithm proposed in this article may require regeneration only in the event of a significant change in the beam energy spectrum, such as beam commissioning.

Finally, should be mentioned that in this study it takes about 3 min to perform a picket fence field and about 50 min to complete a data collection. However, once the algorithm is established, the MLC QA work could be completed by performing only one picket fence field test in the later stage.

## 5 CONCLUSIONS

Different data processing schemes could affect the accuracy of the developed algorithms. Our study successfully developed a simple, efficient, highly robust, and sensitive MLC QA algorithm by integrating three independent algorithms. The proposed algorithm could perform MLC leaf position calibration with an accuracy level of 0.1 mm.

## AUTHOR CONTRIBUTIONS

Chenlu Liu contributed to the project design, made all measurements, performed the data analysis, and wrote the manuscript. Xiaohua yang and Guoping Shan contributed to the project design, measurements, and data analysis. Binbing Wang, Xue Bai, Xiaolong Chen, and Xiaotong Wang participated in measurements and data analysis. Xiaohua Yang and Guoping Shan initiated this project, contributed to its design, and edited the manuscript. Xiaohua Yang and Guoping Shan oversaw the general progress, and determined the final version of the manuscript.

## CONFLICT OF INTEREST STATEMENT

The authors declare no conflicts of interest.
